# The Association between Molar-Incisor Hypomineralization and Dental Caries with Socioeconomic Status as an Explanatory Variable in a Group of Finnish Children

**DOI:** 10.3390/ijerph15071324

**Published:** 2018-06-25

**Authors:** Emma Wuollet, Sakari Laisi, Satu Alaluusua, Janna Waltimo-Sirén

**Affiliations:** 1 Department of Oral and Maxillofacial Diseases, Faculty of Medicine, University of Helsinki, P.O. Box 41, FI-00014 Helsinki, Finland; sakari.laisi@gmail.com (S.L.); satu.alaluusua@helsinki.fi (S.A.); janna.waltimo@helsinki.fi (J.W.-S.); 2 Department of Oral and Maxillofacial Diseases, Helsinki University Hospital, P.O. Box 670, FI-00029 HUS, Finland

**Keywords:** Molar-Incisor Hypomineralization, enamel defects, dental caries, socioeconomic status

## Abstract

The aim of this study was to investigate if a developmental enamel defect known as Molar-Incisor Hypomineralization (MIH) is associated with dental caries. Socioeconomic status (SES) was examined as a confounding factor between caries and MIH. In this cross-sectional study, 636 children, aged 8 to 13 years, from three towns (two rural areas and one urban area) in Finland were examined for MIH in line with the criteria of the European Academy of Paediatric Dentistry. Caries status for permanent teeth was recorded as decayed, missing and filled teeth (DMFT). Caries experience (DMFT > 0) in the first permanent molars (FPMs) was set as an outcome. SES was determined using a questionnaire completed by parents. The prevalence of MIH was 18.1%. The mean DMFT in FPMs for children with MIH was higher than for their peers, 1.03 ± 1.25 vs. 0.32 ± 0.80 (*p* = 0.000, Mann-Whitney U test). In a multivariate analysis using the generalized linear mixed model where locality, SES, age and MIH were taken into account as caries risk indicators, MIH was the strongest risk indicator of caries in FPMs (Odds Ratio: 6.60, 95% Confidence Interval: 3.83–11.39, *p* = 0.000). According to the study results, children with MIH have a higher risk for dental caries than children without MIH.

## 1. Introduction

Caries rates have been declining during the past decades, and many Western populations are classified as low-caries populations. Caries control, however, remains a challenge. The distribution of caries is skewed, and a small number of individuals experience most of the caries lesions and restorations. Therefore, individuals with a high risk for caries need to be identified and treatments carried out respectively. Many caries risk factors have been identified, and one of them is low socioeconomic status (SES) [1]. Some of the risk factors are still under discussion. In a review and meta-analysis on the association between developmental defects of enamel (DDE) and caries, it was concluded that enamel defects may be considered as a potential risk factor for caries [2].

A specific type of DDE, Molar-Incisor Hypomineralization (MIH), is diagnosed when the first permanent molars (FPMs) display areas of poorly mineralized enamel, called hypomineralization [3]. The permanent incisors are frequently affected as well, though canines or other teeth are affected more rarely. The enamel development consists of two major stages: The secretory stage, when the enamel matrix is produced, and the maturation stage, when the mineralization dominates. Between these two stages is a short transitional stage. Enamel defects result from genetic or environmental factors impairing the enamel development. A disturbance to enamel-producing cells, ameloblasts, may influence the cells at any stage, but it has been suggested that ameloblasts are most vulnerable at the transitional and early maturation stages [4]. The enamel mineralization of the FPMs begins around birth and the crown formation is completed around the age of three years [5]. In the incisors, the mineralization begins a few months after birth and is completed somewhat later than in the FPMs [6]. These early developing permanent teeth normally erupt at the age of six to seven years. This is when the enamel surface becomes visible and is exposed to an oral, eventually cariogenic, environment. Clinically, the severity of the MIH defects varies from a mild change in colour (creamy white or yellow opacities) to a severe enamel breakdown [3]. The MIH-associated opaque patches are well-demarcated in contrast to opacities with more diffuse borders, like defects originating from chronic exposure to excess fluoride [3]. Hereditary enamel defects, like the different forms of amelogenesis imperfecta, affect all or majority of permanent (and primary) teeth and should not be confused with MIH.

MIH, especially in its severe form, is often problematic both for the patient and for the dentist. Hypomineralized enamel is soft and porous, and teeth are sensitive to thermal changes, possibly due to the underlying pulpal inflammation [7,8]. Disorganized enamel often breaks down after tooth eruption under the influence of masticatory forces. Restorative treatment is challenging due to difficulties in achieving adequate anesthesia, and because of the poor adhesion of filling materials to hypomineralized enamel [9,10].

Depending on the region/country studied and diagnostic criteria used, the documented prevalence figures for MIH have markedly varied, from 2.9% to 44% [11]. According to a recent systematic review and meta-analysis, the mean global prevalence was 13.1% [12]. Medical problems during the pre- and postnatal periods and early childhood and exposure to environmental toxicants have continuously arisen as possible etiologic factors [13,14,15,16]. Research has also been done to try to find a possible genetic link to MIH [17,18,19]. Thus, it seems that the condition is multifactorial. Evidence is, however, still too weak to draw any definitive conclusion about the etiology of MIH.

Another subject of many studies has been the role of MIH as a risk factor for dental caries. According to a recent systematic review, most studies reported a positive correlation between MIH and caries, but the quality of the studies was compromised, and a need was expressed for more well-designed studies [20]. Most of the studies did not evaluate possible confounding factors between MIH and caries. MIH defects develop in early childhood, when child’s health and nutritional status may be affected by SES of the family. Moreover, SES plays a role in the etiology of caries. Because caries and MIH may share risk factors, it is important that possible confounding factors are considered. A possible confounding factor between DDE and caries is SES [2].

The aim of this study was to evaluate the association between MIH and caries taking into account the effect of SES. The study hypothesis was that MIH is associated with caries in FPMs regardless of SES. 

## 2. Study Design

### 2.1. Study Population 

The participants in this cross-sectional study were 8- to 13-year-old children attending public comprehensive schools in three regions in Finland. The biggest comprehensive schools from two rural towns, Lammi and Jalasjärvi, and from the city of Lappeenranta, were selected, and the pupils in the 2nd to 5th grades, 994 children in total, were invited to join the study. The children were born in 1990–1996. The parents (usually the mother) of 676 children (68%) gave informed consent for the participation of the children in the clinical examination and filled out a questionnaire related to SES, health, and the way of living of the family and the child. Inclusion criteria were as follows: signed informed consent, age between 8 and 13 years, all FPMs erupted, and no fixed orthodontic appliances interfering with teeth evaluation. The final number of participants was 636 (64% of the invited children). 

In the Lammi region, the fluoride content in communal pipe water is <0.1 mg/L and in wells in the sparsely populated areas it is mostly <0.2 mg/L. In Jalasjärvi, the fluoride content in communal pipe water is 0.5–1 mg/L, and in Lappeenranta approximately 0.25 mg/L. 

### 2.2. Clinical Examination

The clinically experienced participating dentist (Sakari Laisi) was trained for the screening of MIH, diffuse opacities and enamel hypoplasia with Satu Alaluusua a professor of paediatric dentistry, until consensus was obtained. MIH was screened in line with the criteria of the European Academy of Paediatric Dentistry (EAPD) [21], and The World Dental Federation (FDI) criteria for DDE were used for screening of diffuse opacities and enamel hypoplasia [22]. After the training phase and screening of 21 subjects, calibration was done and the inter-examiner kappa score between Sakari Laisi and Satu Alaluusua for teeth with DDE was 0.96, and for classified defects (MIH, diffuse opacity and hypoplasia) it was 0.81. Repeatability of the recordings of Sakari Laisi was tested by examining the dentitions of a sample of children twice at an interval of a few weeks. The intra-examiner kappa scores were 0.91 for DDE and 0.90 for classified defects. The inter-examiner and intra-examiner kappa scores for decayed teeth were 0.93 and 0.88, respectively.

The dental examinations were performed in the dental clinic under standard dental lighting. The same calibrated dentist (Sakari Laisi) performed all the examinations using a mirror and a periodontal probe.

FPMs and permanent incisors were examined wet for MIH-characteristic hypomineralization defects i.e., demarcated opacities, post-eruptive breakdown of the hypomineralized enamel, and atypical form of a restoration/caries. These criteria are in line with the judgement criteria for MIH set by the European Academy of Paediatric Dentistry (EAPD) in 2003 [21]. Since the reproducibility of screening small demarcated opacities is low [23], only lesions with a diameter of 2 mm or more were included. This is in line with earlier studies [24,25,26]. The diagnosis of MIH was set when a lesion was present in at least one FPM. In line with the EAPD criteria for MIH, enamel hypoplasia, diffuse opacities indicating fluorosis, and developmental enamel defects affecting the majority or all teeth were not recorded as MIH.

Caries status was recorded in permanent teeth using the DMFT index (number of Decayed, Missing or Filled Teeth). Visual investigation enhanced by fiber-optic transillumination was used to diagnose caries. Initial caries lesions, teeth extracted due to orthodontic reasons and congenitally missing teeth were not included in the DMFT value. The diagnostic criteria for caries were in line with those of the WHO [27]. Developmental defects or caries in primary teeth were not recorded.

### 2.3. Questionnaire

The questionnaire used in the study was originally designed for the purpose of studying associations between allergies and lifestyle (a mother-child research questionnaire by the National Institute for Health and Welfare, Kuopio, Finland). It was supplemented with questions relevant to the present study. Two variables were used to define the SES of the family:

- Family income: The question about annual family gross income had six classes, from the lowest, less than 13,500€, to the highest, more than 50,400€. For further analysis, to yield a more informative distribution of the sample, the lowest three classes were combined, resulting in a total of four categories.
- Education of the mother: The question about mother’s education had four classes: primary and secondary school, vocational school or equivalent, high school or higher vocational school and college/university graduate. The first level also included former forms of the 9-year-long Finnish basic education.

The questionnaire was filled by the parent/parents at home in advance and returned at the examination. The examiner was blinded to the information given in the questionnaire.

### 2.4. Statistical Analysis

The statistical analyses were conducted using IBM SPSS version 24.0 (IBM Corp., Armonk, NY, USA), and included descriptive statistics of MIH on tooth level, caries and SES factors, including normality tests and χ^2^-test of independence. To test the study hypothesis, the outcome variable was determined by following variables: the DMFT value in FPMs, ranging from 0 to 4, and the caries experience in FPMs (DMFT = 0 vs. DMFT > 0). MIH was determined as an exposure variable. The two SES factors (family income and mother’s education level) were taken into account as possible confounding factors. Also, analyses were adjusted to age and region. 

The Kruskal-Wallis 1-way ANOVA test was used to test the statistical difference in the DMFT value in FPMs between children with and without MIH. The association of SES factors and caries experience in FPMs (DMFT > 0) was tested using χ^2^-test. The distributions of the study variables between three regions were tested using χ^2^-test and Kruskal-Wallis 1-way ANOVA test. In case of statistically significant association, the Bonferroni method was used for adjusted p-values in pairwise comparisons.

To test the association between the outcome (caries experience in FPMs) and exposure variable (MIH) taking into account the confounding factors (SES, age and region), the generalized linear mixed model analysis with logit link was conducted. Different models were conducted attempting to alternately include one of the SES variables, but a model including both SES variables was found to be most fitting. Adjusted odds ratios (ORs) and 95% confidence intervals (CI) were calculated to test whether MIH or confounding factors were associated with elevated odds of having caries relative to being caries-free. Statistical significance level was set at *p* = 0.05 (two-tailed).

### 2.5. Study Ethics

Parents of the children approved the participation of the children in the clinical examination and filled out a questionnaire. Clinical examinations were part of the dental check-ups that were offered to everyone regardless of the decision to participate in the study. Radiography was not used for caries diagnostics. The basic service public utility federation of municipalities JIK (Jalasjärvi, Ilmajoki, Kurikka), the Lammi-Tuulos federation of municipalities, and the Ethics Committee at South Karelia Hospital District, Lappeenranta, Finland, approved the study (A02/03).

## 3. Results

A total of 636 children were included in the study. Distribution of the study variables between children with and without MIH are shown in Table 1.

There were 115 children with MIH (18.1%). The total number of FPMs with an MIH defect was 225. The mean number of MIH-affected FPMs among children with MIH was 2.0 ± 1.1. MIH was more often diagnosed in the maxillary FPMs (*n* = 143) than in the mandibular FPMs (*n* = 82) (63.6% vs. 36.4%). 

There were significantly less children with MIH in Jalasjärvi than in Lappeenranta (Table 1). Other variables also differed between regions. Children from Lammi had smaller DMFT values than children from Jalasjärvi or Lappeenranta (0.38 vs. 0.66 and 0.68, adjusted *p* = 0.050 and 0.011, respectively, in pairwise comparisons, Kruskal Wallis 1-way ANOVA test). Children from Jalasjärvi were younger than children from Lammi or Lappeenranta (mean age 10.1 vs. 10.8 and 10.7, adjusted *p* = 0.000 in both pairwise comparisons, Kruskal Wallis 1-way ANOVA test). In Lappeenranta, a significantly higher proportion of children belonged to the highest mother’s education and family income groups than in Lammi or Lappeenranta (*p* = 0.000, χ^2^-test).

The prevalence of caries was 27.8% (*n* = 177 children) for all permanent teeth (DMFT > 0). For FPMs, the prevalence of caries was 24.0% (*n* = 153 children) (DMFT > 0 in FPMs). Of the FPMs affected by MIH, 24.8% had atypical restorations or demarcated opacities with caries and were deduced to be affected by caries. This was more prevalent in the mandibular (28 out of 82) than in the maxillary (28 out of 143) FPMs with MIH (34.1% vs. 19.6%).

**Figure 1:**
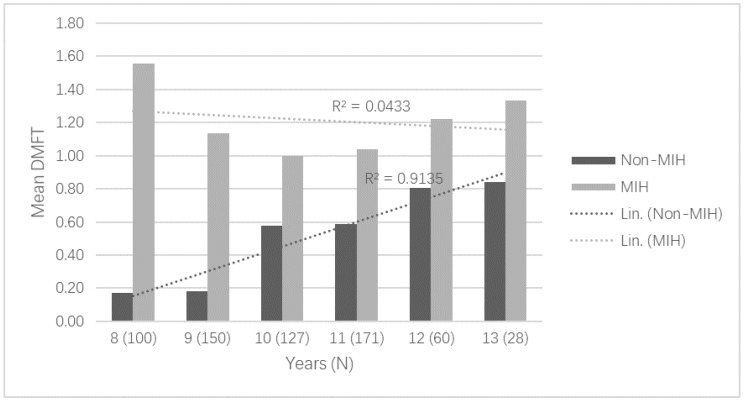
Mean DMFT in permanent teeth in the cross-sectional cohort sub-grouped by age (years) and presence/absence of Molar-Incisor Hypomineralization (MIH). The number of children in each age group is given in parentheses.

In the univariate analysis, MIH was associated with caries in FPMs. Of the children with MIH, 52.2% presented caries in FPMs compared to 17.9% of the children without MIH (OR 5.02, 95% CI 3.27–7.71). To assess the pattern of caries development, the cross-sectional sample was sub-grouped by age and DMFT values were studied for MIH and non-MIH children in every age group. The mean DMFT increased by age among non-MIH children, whereas among MIH children the mean DMFT in age groups from 8 to 13 years stayed more constant (Figure 1). 

In the univariate analysis, family income was associated with caries in FPMs, so there were significantly more caries affected children in the lowest than in higher income groups (*p* = 0.034, χ^2^-test). Mother’s education level was associated with caries so caries experience decreased from lower to higher education levels (*p* = 0.049, χ^2^-test). 

In a generalized mixed model analysis with caries in FPMs as an outcome variable, where locality, age, and both SES variables were taken into account, MIH was associated with caries with the OR of 6.6 compared to the children without MIH. Other significant caries risk indicators were age and location (Table 2). Family income was associated with caries, so the estimated odds for having caries in FPMs were higher in the lowest income group than in the second highest income group (Table 2), but the mother’s education level was not associated with caries in FPMs.

## 4. Discussion

This cross-sectional epidemiological study was carried out to explore whether the presence of a developmental enamel defect, Molar-Incisor Hypomineralization (MIH) increases the child’s risk for dental caries, taking into consideration the socioeconomic status (SES) of the child as a confounding factor. MIH was associated with caries in permanent teeth with the adjusted odds ratio of more than 6-fold compared to the children without MIH. This result is in line with a recent case-control study from Brazil [28].

Of the 636 Finnish 8- to 13-year-old children included in the study, 18.1% had one or more FPMs with enamel hypomineralization i.e., MIH. The diagnostic criteria for MIH were in line with those set by the EAPD in 2003 [21], but lesions smaller than 2 mm in diameter were not recorded. The children were residents of three different regions that turned out to differ in the prevalence of MIH. All participating children were examined by the same investigator. No gender differences were found between regions. There were significantly more children with MIH in Lappeenranta than in Jalasjärvi or Lammi. The children from Jalasjärvi were slightly younger than children from Lammi or Lappeenranta, but since MIH was equally prevalent at all ages from 8 to 13 years, the observed regional differences in the prevalence of MIH should be real. Lappeenranta is an industrial town located in Eastern Finland. It has more residents than Jalasjärvi and Lammi, which are classified as rural towns. It was seen that family income and mother’s education level were lower in rural areas than in an urban area. Fluoride content in drinking water varied between regions, so that it was higher in Jalasjärvi than in Lammi or Lappeenranta. Because of several possible sources of fluoride (water, tooth paste, supplements, dental professionals), it was not possible to assess true exposure to fluoride. Variations in the prevalence of MIH between regions have also been detected in Northern England [29] and in Germany [30]. In England, similarly to our study, the prevalence of MIH was lower in a fluoridated area than in the rest of the region [29]. The prevalence differences and possible etiological factors of MIH, such as medical problems in early childhood have been discussed more in earlier papers by the authors [15,31].

DMFT values both in all permanent teeth and in FPMs were higher among children with MIH than among their peers. In the multivariate analysis, the association of MIH and caries was calculated. The model was conducted taking into account that both variables, as well as the factors defining SES, varied between the three regions. MIH was the strongest caries risk indicator. As it can be supposed, age increased the risk of caries. Interestingly, fewer children in Lammi than in Jalasjärvi or Lappeenranta were affected by caries. The reason for this remains unknown. Overall, 28% of the subjects were affected by caries in permanent teeth and 24% were affected by caries in FPMs. In a low-caries population, and in young individuals, developmental defects are less likely to be masked by caries, and atypical restorations can be recognized.

Maxillary FPMs were more frequently affected by MIH than mandibular FPMs. This is in line with an earlier Finnish study [26]. Demarcated enamel opacities in mandibular FPMs were, however, more frequently affected by caries than opacities in maxillary FPMs. The reason for this finding could be that mandibular molars erupt earlier than maxillary molars. Thus, because of the cross-sectional study design, the caries follow-up period was longer for mandibular molars than maxillary molars.

In general, the present findings of the association between MIH and caries are in line with those from earlier studies. Children with MIH have been reported to have more caries affected teeth (represented by the DMFT value) than their peers [26,28,30,32], or caries has been more prevalent among children with MIH [32,33,34]. One study did not find differences in DMF scores between MIH and non-MIH children [35], but in that study atypical restorations were not included in the DMF scores, and therefore the prevalence of caries among MIH children may have been underestimated. In our study, atypical restorations were also recorded in the DMFT value.

Morphological analyses of MIH teeth show that the hypomineralized enamel is less organized, is porous, and has a higher organic content than normal enamel [4,8,36]. The decreased mechanical properties can lead to enamel breakdown which will expose dentine to oral bacteria. SEM images of the surface of opacity and hypoplastic enamel show areas of disruption of surface integrity; these areas would promote colonization of cariogenic bacteria, such as mutans streptococci [37]. In histologic analysis of MIH teeth, bacterial penetration has been found deep in clinically intact hypomineralized enamel [38]. It is possible that due to increased sensitivity of hypomineralized teeth, a proper level of dental hygiene cannot be maintained, leading to higher caries rates. In this study, caries experience was not examined between MIH and non-MIH teeth of the same patient. It was recently reported, however, that the teeth clinically affected by MIH are more susceptible for caries than non-affected teeth of the same patient [28].

Vargas-Ferreira et al. conducted a meta-analysis on caries and developmental defects of enamel (DDE) and found a positive association between the two [2]. The authors noted, however, that most studies have not considered the effect of SES. Low SES is associated with a higher risk of caries [1], and a disadvantaged background could also be a risk factor for developmental defects. For instance, a low birth weight has been found to increase the risk of enamel hypoplasia [39] and low birth weight occurs more frequently among those from a lower SES [40]. An association has been found between low birth weight and MIH as well [13]. In the present study, SES did not affect the association between MIH and caries in FPMs. Compared to SES, MIH was a greater risk indicator for caries in FPMs. This finding suggests that the basic caries-controlling methods, such as brushing with fluoridated tooth paste and maintaining a low-sugar diet, are not sufficient to keep a dentition with hypomineralization defects intact. Moreover, as can be concluded from the present study, children with MIH seem to be affected by caries early. Their FPMs were affected by caries already at ages 8 and 9 years, while this is seldom the case in children without MIH. Therefore, routine, early caries examinations among all children with FPMs are highly recommended.

Americano et al. [20] conducted a review on the association of MIH and caries and used the Newcastle-Ottawa Scale (NOS) “star system” [41] to assess the quality of studies. Most of the studies included in the review achieved three stars in an eight-star rating. Our study fills most of the quality criteria used in the review and would achieve six stars. The reason for not achieving the full eight stars was that the assessment of caries and MIH was not blinded, as the examiner recorded both at the same time. Separate charts were used, however, to record caries/fillings and DDE. Another limitation to note is that some of the children already had full permanent dentition and the status of caries in primary teeth remained unknown. To avoid bias, caries recordings in primary teeth were left outside the present analysis.

Although the study sample was not randomly chosen, the results can, with some precaution, be generalized to the population. The participants were recruited from public schools which are the only choice of schooling in most of the towns in Finland. The setting resembles a population-based study, though participation rate was reduced and may have led to skewed distribution of SES, resulting a sample with more children from higher SES families than in the background population. 

## 5. Conclusions

This study supports the findings of earlier studies that MIH increases the risk of caries. Taking into account the regional differences, MIH, in comparison to SES, was a greater risk indicator for caries in FPMs. The FPMs of children with MIH were more often affected by caries than FPMs of their peers. Especially in a low-caries risk population such as in Finland, the impact of MIH on caries development should be considered. Besides, because the treatment of caries in hypomineralized teeth is challenging, children with MIH should be considered to have a high risk for caries, regardless of their socioeconomic status, and offered versatile caries-preventive measures as well as frequent follow-up visits to dental care. 

## Figures and Tables

**Table 1:** Distribution of study variables between children with and without Molar-Incisor Hypomineralization (MIH).

Variable		MIH	Non-MIH	Total	*p*-Value
Children		115	521	636	
Region	Lammi	32 (27.8)	156 (29.9)	188 (29.6)	0.000
	Jalasjärvi	15 (13.0) *	172 (33.0)	187 (29.4)	
	Lappeenranta	68 (59.1) *	193 (37.0)	261 (41.0)	
DMFT value		1.17 ± 1.39	0.46 ± 1.12	0.59 ± 1.21	0.000 ^a^
DMFT in FPMs		1.03 ± 1.25	0.32 ± 0.80	0.45 ± 0.94	0.000 ^a^
Age, year		10.5 ± 1.4	10.5 ± 1.3	10.5 ± 1.4	0.848 ^a^
Gender	Girls	57 (49.6)	250 (48.0)	307 (48.3)	0.094
	Boys	58 (50.4)	271 (52.0)	329 (51.7)	
Family income, €	<25,200	16 (16.7) *	117 (27.0)	133 (25.1)	0.037
	25,200–33,600	15 (15.6)	90 (20.8)	105 (19.8)	
	33,600–50,400	32 (33.3)	123 (28.4)	155 (29.3)	
	>50,400	33 (34.4) *	103 (23.8)	136 (25.7)	
Mother’s education	Prim. & second. school	20 (17.5)	74 (14.5)	94 (15.0)	0.357
	Vocational school	29 (25.4)	146 (28.5)	175 (28.0)	
	High school/Higher vocational school	46 (40.4)	232 (45.3)	278 (44.4)	
	University	19 (16.7)	60 (11.7)	79 (12.6)	

Data are presented as *n* (%) or mean ± SD. MIH: Molar-Incisor Hypomineralization. FPMs: First Permanent Molars. DMFT: Decayed, Missing or Filled Teeth in permanent teeth. *p*-values from χ^2^ or Kruskal Wallis 1-way ANOVA test, * -marked column proportions differ significantly from each other (*z*-test with Bonferroni method). ^a^ *p*-values were obtained by Kruskal Wallis 1-way ANOVA test and refer to differences in mean rank instead of reported means.

**Table 2:** The generalized linear mixed model analysis of caries in FPMs (DMFT = 0/DMFT > 0) as a dependent variable and its risk indicators as covariates.

Variable	Categories	Coefficient	OR (95% CI)	*p*-Value
MIH	MIH	1.88	6.60 (3.83–11.39) **	0.000
Age of the child	Years	0.25	1.29 (1.08–1.53) **	0.004
Region	Lammi (reference)	0	1	
	Jalasjärvi	1.29	3.62 (1.67–7.87) **	0.001
	Lappeenranta	0.88	2.41 (1.16–4.99) *	0.018
Family income, €	<25,200 (reference)	0	1	
	25,200–33,600	−0.50	0.61 (0.30–1.25)	0.177
	33,600–50,400	−0.93	0.39 (0.20–0.78) **	0.008
	>50,400	−0.47	0.63 (0.30–1.32)	0.217
Mother’s education	Prim. & second. school (reference)	0	1	
	Vocational school	0.58	1.79 (0.79–4.07)	0.166
	High school/Higher vocational school	−0.0.3	0.97 (0.42–2.22)	0.944
	University	0.06	1.06 (0.38–2.96)	0.909

The generalized linear mixed model with logit link. The model has its own random slopes socioeconomic status (SES) variables in relation to locality, as well as its own intercept for each subject. The exponential coefficient is presented as ORs. No. of cases included in the analysis: 525. Information criterion, Akaike Corrected was 2530.765. * *p* < 0.05, ** *p* < 0.01. MIH: Molar-Incisor Hypomineralization.

## References

[1] Schwendicke F., Dorfer C.E., Schlattmann P., Foster P.L., Thomson W.M., Paris S. (2015). Socioeconomic inequality and caries: A systematic review and meta-analysis. J. Dent. Res..

[2] Vargas-Ferreira F., Salas M.M.S., Nascimento G.G., Tarquinio S.B.C., Faggion C.M., Peres M.A., Thomson W.M., Demarco F.F. (2015). Association between developmental defects of enamel and dental caries: A systematic review and meta-analysis. J. Dent..

[3] Weerheijm K.L., Jalevik B., Alaluusua S. (2001). Molar-incisor hypomineralisation. Caries Res..

[4] Jalevik B., Norén J.G. (2000). Enamel hypomineralization of permanent first molars: A morphological study and survey of possible aetiological factors. Int. J. Paediatr. Dent..

[5] Nystrom M.E., Ranta H.M., Peltola J.S., Kataja J.M. (2007). Timing of developmental stages in permanent mandibular teeth of Finns from birth to age 25. Acta Odontol. Scand..

[6] Reid D.J., Dean M.C. (2006). Variation in modern human enamel formation times. J. Hum. Evol..

[7] Rodd H.D., Morgan C.R., Day P.F., Boissonade F.M. (2007). Pulpal expression of TRPV1 in molar incisor hypomineralisation. Eur. Arch. Paediatr. Dent..

[8] Fagrell T.G., Dietz W., Jalevik B., Noren J.G. (2010). Chemical, mechanical and morphological properties of hypomineralized enamel of permanent first molars. Acta Odontol. Scand..

[9] Kotsanos N., Kaklamanos E.G., Arapostathis K. (2005). Treatment management of first permanent molars in children with Molar-Incisor Hypomineralisation. Eur. J. Paediatr. Dent..

[10] Lygidakis N.A., Wong F., Jalevik B., Vierrou A.M., Alaluusua S., Espelid I. (2010). Best Clinical Practice Guidance for clinicians dealing with children presenting with Molar-Incisor-Hypomineralisation (MIH): An EAPD Policy Document. Eur. Arch. Paediatr. Dent..

[11] Elfrink M.E., Ghanim A., Manton D.J., Weerheijm K.L. (2015). Standardised studies on Molar Incisor Hypomineralisation (MIH) and Hypomineralised Second Primary Molars (HSPM): A need. Eur. Arch. Paediatr. Dent..

[12] Schwendicke F., Elhennawy K., Reda S., Bekes K., Manton D.J., Krois J. (2018). Global burden of molar incisor hypomineralization. J. Dent..

[13] Brogardh-Roth S., Matsson L., Klingberg G. (2011). Molar-incisor hypomineralization and oral hygiene in 10- to-12-yr-old Swedish children born preterm. Eur. J. Oral Sci..

[14] Tourino L.F., Correa-Faria P., Ferreira R.C., Bendo C.B., Zarzar P.M., Vale M.P. (2016). Association between molar incisor hypomineralization in schoolchildren and both prenatal and postnatal factors: A population-based study. PLoS ONE.

[15] Wuollet E., Laisi S., Salmela E., Ess A., Alaluusua S. (2016). Molar-incisor hypomineralization and the association with childhood illnesses and antibiotics in a group of Finnish children. Acta Odontol. Scand..

[16] Babajko S., Jedeon K., Houari S., Loiodice S., Berdal A. (2017). Disruption of steroid axis, a new paradigm for molar incisor hypomineralization (MIH). Front. Physiol..

[17] Jeremias F., Koruyucu M., Kuchler E.C., Bayram M., Tuna E.B., Deeley K., Pierri R.A., Souza J.F., Fragelli C.M., Paschoal M.A. (2013). Genes expressed in dental enamel development are associated with molar-incisor hypomineralization. Arch. Oral Biol..

[18] Jeremias F., Pierri R.A., Souza J.F., Fragelli C.M., Restrepo M., Finoti L.S., Bussaneli D.G., Cordeiro R.C., Secolin R., Maurer-Morelli C.V. (2016). Family-Based Genetic Association for Molar-Incisor Hypomineralization. Caries Res..

[19] Kuhnisch J., Thiering E., Heitmuller D., Tiesler C.M., Grallert H., Heinrich-Weltzien R., Hickel R., Heinrich J. (2014). GINI-10 Plus Study Group; LISA-10 plus study group. Genome-wide association study (GWAS) for molar-incisor hypomineralization (MIH). Clin. Oral Investig..

[20] Americano G.C., Jacobsen P.E., Soviero V.M., Haubek D. (2017). A systematic review on the association between molar incisor hypomineralization and dental caries. Int. J. Paediatr. Dent..

[21] Weerheijm K.L., Duggal M., Mejare I., Papagiannoulis L., Koch G., Martens L.C., Hallonsten A.L. (2003). Judgement criteria for molar incisor hypomineralisation (MIH) in epidemiologic studies: A summary of the European meeting on MIH held in Athens, 2003. Eur. J. Paediatr. Dent..

[22] FDI Working Group (1992). A review of the developmental defects of enamel index (DDE Index). Commission on oral health, research & epidemiology. Report of an FDI Working Group. Int. Dent. J..

[23] Sucking G.W., Brown R.H., Herbison G.P. (1985). The prevalence of developmental defects of enamel in 696 nine-year-old New Zealand children participating in a health and development study. Community Dent. Health.

[24] Calderara P.C., Gerthoux P.M., Mocarelli P., Lukinmaa P.L., Tramacere P.L., Alaluusua S. (2005). The prevalence of molar incisor hypomineralisation (MIH) in a group of Italian school children. Eur. J. Paediatr. Dent..

[25] Jalevik B., Klingberg G., Barregard L., Noren J.G. (2001). The prevalence of demarcated opacities in permanent first molars in a group of Swedish children. Acta Odontol. Scand..

[26] Leppäniemi A., Lukinmaa P.L., Alaluusua S. (2001). Nonfluoride hypomineralizations in the first molars and their impact on treatment need. Caries Res..

[27] World Health Organization (1997). Oral Health Surveys: Basic Methods.

[28] Grossi J.A., Cabral R.N., Leal S.C. (2017). Caries experience in children with and without molar-incisor hypomineralisation: A case-control study. Caries Res..

[29] Balmer R., Toumba J., Godson J., Duggal M. (2012). The prevalence of molar incisor hypomineralisation in Northern England and its relationship to socioeconomic status and water fluoridation. Int. J. Paediatr. Dent..

[30] Petrou M.A., Giraki M., Bissar A.R., Basner R., Wempe C., Altarabulsi M.B., Schäfer M., Schiffner U., Beikler T., Schulte A.G. (2014). Prevalence of molar-incisor-hypomineralisation among school children in four German cities. Int. J. Paediatr. Dent..

[31] Wuollet E., Laisi S., Salmela E., Ess A., Alaluusua S. (2014). Background factors of molar-incisor hypomineralization in a group of Finnish children. Acta Odontol. Scand..

[32] Jeremias F., de Souza J.F., Silva C.M., Cordeiro R.C., Zuanon A.C., Santos-Pinto L. (2013). Dental caries experience and molar-incisor hypomineralization. Acta Odontol. Scand..

[33] Da Costa-Silva C.M., Jeremias F., de Souza J.F., Cordeiro R.C., Santos-Pinto L., Zuanon A.C. (2010). Molar incisor hypomineralization: Prevalence, severity and clinical consequences in Brazilian children. Int. J. Paediatr. Dent..

[34] Bhaskar S., Hedge S. (2014). Molar-incisor hypomineralization: Prevalence, severity and clinical characteristics in 8- to 13-year-old children of Udaipur, India. J. Indian Soc. Pedod. Prev. Dent..

[35] Heitmuller D., Thiering E., Hoffmann U., Heinrich J., Manton D., Kuhnisch J., Neumann C., Bauer C.P., Heinrich-Weltzien R., Hickel R. (2013). Is there a positive relationship between molar incisor hypomineralisations and the presence of dental caries?. Int. J. Paediatr. Dent..

[36] Xie Z.H., Mahoney E.K., Kilpatrick N.M., Swain M.V., Hoffman M. (2007). On the structure-property relationship of sound and hypomineralized enamel. Acta Biomater..

[37] Caufield P.W., Li Y., Bromage T.G. (2012). Hypoplasia-associated severe early childhood Caries-a proposed definition. J. Dent. Res..

[38] Fagrell T.G., Lingstrom P., Olsson S., Steiniger F., Noren J.G. (2008). Bacterial invasion of dentinal tubules beneath apparently intact but hypomineralized enamel in molar teeth with molar incisor hypomineralization. Int. J. Paediatr. Dent..

[39] Nelson S., Albert J.M., Geng C., Curtan S., Lang K., Miadich S., Heima M., Malik A., Ferretti G., Eggertsson H. (2013). Increased enamel hypoplasia and very low birthweight infants. J. Dent. Res..

[40] Hamilton B.E., Martin J.A., Ventura S.J. (2012). Births: Preliminary data for 2011. Natl. Vital Stat. Rep..

[41] Wells G., Shea B., O’Connell D., Peterson J., Welch V., Losos M., Tugwell P. (2016). The Newcastle-Ottawa Scale (NOS) for Assessing the Quality of Nonrandomized Studies in Meta-Analyses. http://www.ohri.ca/programs/clinical_epidemiology/oxford.asp.

